# Selective Inhibition of Insulin-Degrading Enzyme Eliminates Hemolysis Interference in Serum Insulin Measurements

**DOI:** 10.3390/diagnostics16121927

**Published:** 2026-06-22

**Authors:** María Rodríguez-García, Bernardino González de la Presa, Aleix B. Fabregat-Bolufer, Naira Rico, Helena Castella, Alejandro Calvera-Rayo, Marga Giménez, Felicia A. Hanzu, Manuel Morales-Ruiz, Gregori Casals

**Affiliations:** 1 Biochemistry and Molecular Genetics Department, Biomedical Diagnostic Center (CDB), Hospital Clínic of Barcelona, 08036 Barcelona, Spain; mrodriguezg@clinic.cat (M.R.-G.); begonzal@clinic.cat (B.G.d.l.P.); afabregat@clinic.cat (A.B.F.-B.); nrico@clinic.cat (N.R.); hcastella@clinic.cat (H.C.); calvera@clinic.cat (A.C.-R.); morales@clinic.cat (M.M.-R.); 2 Fundació de Recerca Clínic Barcelona-Institut d’Investigacions Biomèdiques August Pi i Sunyer (FCRB-IDIBAPS), 08036 Barcelona, Spain; gimenez@clinic.cat (M.G.); fhanzu@clinic.cat (F.A.H.); 3 Diabetes Unit, Hospital Clínic of Barcelona, 08036 Barcelona, Spain; 4 Endocrinology and Nutrition Department, Hospital Clínic of Barcelona, 08036 Barcelona, Spain; 5 Centro de Investigación Biomédica en Red de Diabetes y Enfermedades Metabólicas Asociadas (CIBERDEM), 28029 Madrid, Spain; 6 Department of Medicine, Faculty of Medicine and Health Sciences, University of Barcelona, 08036 Barcelona, Spain; 7 Department of Biomedicine, Faculty of Medicine and Health Science, University of Barcelona, 08036 Barcelona, Spain; 8 Centro de Investigación Biomédica en Red de Enfermedades Hepáticas y Digestivas (CIBEREHD), 28029 Madrid, Spain; 9 Department of Fundamental and Clinical Nursing, Faculty of Nursing, University of Barcelona, L’Hospitalet de Llobregat, 08907 Barcelona, Spain

**Keywords:** insulin, hemolysis, insulin-degrading enzyme, interference

## Abstract

**Objectives**. Hemolysis significantly interferes with insulin measurements in clinical settings, leading to inaccurate results. Although the activity of insulin-degrading enzyme (IDE) is assumed to be the primary mechanism, the potential involvement of additional mechanisms remains unclear. This study aims to determine if IDE is the sole cause of this interference by using a selective IDE inhibitor, 6bK, and to explore whether this inhibition can completely prevent hemolysis-related inaccuracies in insulin assays. **Methods**. The effects of 6bK on insulin degradation were evaluated in hemolyzed and non-hemolyzed serum samples at room temperature, following the CLSI guidelines EP07-A2 and C56-A. Insulin levels were measured using chemiluminescent immunoassays. Additional assessments included the impact of 6bK on serum C-peptide, proinsulin, and standard biochemical parameters. The effects of 6bK were also evaluated at 4 °C and after 21 days of storage at room temperature prior to use. **Results**. Hemolysis caused a significant decrease in insulin concentrations, dependent on hemolysate levels and incubation time. The addition of 10 µM 6bK completely reversed hemolysis-induced insulin degradation in serum across a broad range of insulin baseline concentrations and degrees of hemolysis. Furthermore, 6bK did not affect insulin levels in non-hemolyzed samples or alter the quantification of C-peptide, proinsulin, or standard biochemical parameters. **Conclusions**. The decrease in serum insulin concentration due to hemolysis is exclusively attributed to the action of IDE. Selective inhibition of IDE by 6bK effectively eliminates hemolysis-induced interference in insulin measurements, providing a novel and reliable solution for accurate insulin quantification in hemolyzed clinical samples.

## 1. Introduction

The first immunoassay developed was a radioimmunoassay for insulin [1], enabling the sensitive and specific quantification of this hormone, which significantly contributed to our current understanding of diabetes [2]. The interfering effect of hemolysis in serum insulin determination was rapidly identified and linked to the release of intraerythrocytic enzymes [3,4,5,6,7]. Subsequently, the activity of the insulin-degrading enzyme (IDE) [8] has been associated with this interference.

Insulin measurements are valuable in various clinical settings, including the investigation of hypoglycemia, insulin resistance (including indices such as HOMA-IR or QUICKI [9]), and insulin resistance-related conditions (e.g., polycystic ovary syndrome). They are also incorporated into some algorithms for assessing liver fibrosis. Currently, most laboratories use chemiluminescent immunoassays, which are also affected by hemolysis-related interference [10,11]. This limits their clinical utility, as even mild hemolysis can significantly impact results [12]. Furthermore, many scenarios involving insulin measurements, such as neonatal hypoglycemia and serial catheter samples from oral or intravenous glucose tolerance tests, are particularly prone to hemolysis [13,14].

Although IDE is presumed to be the primary cause of the interference [8,15], the lack of specific inhibitors has hindered its confirmation. In the current study, we assessed the effects of 6bK, a selective IDE inhibitor identified and exhaustively characterized by Maianti et al. [16], in serum hemolyzed samples. Furthermore, we assessed whether the use of this inhibitor could be an effective strategy for preventing interference in insulin determination caused by hemolysis in clinical laboratories. The results demonstrate that the interference in insulin determination due to hemolysis is solely attributable to the action of IDE, and that its selective inhibition by 6bK enables the accurate quantification of this hormone in hemolyzed samples.

## 2. Materials and Methods

### 2.1. Chemical Reagents and Laboratory Assays

The 6bK was obtained from Tocris Bioscience (Bristol, UK), and a stock solution was prepared in water at 1 mM. Bacitracin was obtained from Merck (Darmstadt, Germany), with a stock solution also prepared in water at 70 mM. Insulin levels were quantified using chemiluminescence immunoassays run on the IM1600 Atellica analyzer (Siemens Healthineers, Tarrytown, NY, USA) and the Roche Cobas 6000 analyser (Roche Diagnostics Corp., Indianapolis, IN, USA). Hemoglobin concentrations were measured using an Advia 2120i analyzer (Siemens Healthineers, Tarrytown, NY, USA). The HIL index and standard biochemical parameters (sodium, potassium, chloride, calcium, magnesium, glucose, creatinine, bilirubin, total proteins, albumin, C-reactive protein, aspartate aminotransferase, alanine aminotransferase, gamma-glutamyl transferase, lactate dehydrogenase, lipase, alkaline phosphatase, and amylase) were measured on the Atellica CH analyzer (Siemens Healthineers, Tarrytown, NY, USA). C-peptide was measured by chemiluminescence immunoassay run on the Immulite 2000 analyzer (Siemens Healthineers, Tarrytown, NY, USA), and proinsulin was measured by ELISA (Zentech, Angleur, Liege, Belgium).

### 2.2. Evaluation of the Effect of Hemolysis on Serum Insulin Measurements

The effects of hemolysis on insulin measurements were evaluated following the CLSI guidelines EP07-A2 [17] and C56-A [18]. Serum samples without visible hemolysis were pooled to create a base serum pool. A hemolysate with an Hb concentration of 100 g/L (10,000 mg/dL) was prepared from 5 mL of heparinized blood. First, blood was centrifuged at 1200× *g* for 10 min. The plasma was removed and replaced with 10 mL of isotonic saline solution. The tube was inverted several times, followed by centrifugation at 1200× *g* for 10 min, and the supernatant was discarded. This washing step was repeated twice. Then, the pelleted cells were resuspended in 2.5 mL of deionized water and mixed thoroughly by inverting the tube. The suspension was frozen at −20 °C for a minimum of 12 h, thawed, and vortexed. Cellular debris was removed by centrifugation at 1200× *g* for 30 min. The supernatant (hemolysate) was collected, and Hb concentration was measured and adjusted to 100 g/L (10,000 mg/dL) using deionized water as diluent. Finally, aliquots from the base non-hemolyzed serum pools were spiked with increasing amounts of hemolysate to prepare serial hemolysis samples, with Hb concentration up to 500 mg/dL The volume of the hemolysate stock solutions added to the base serum pools was below 5%. Insulin concentrations of base serum pools ranged from 5 to 50 mIU/L. Experiments were performed both at room temperature and at 4 °C. Hemolysis was also induced in whole blood by vortexing for 2 min before serum separation.

Insulin concentrations in hemolyzed serum samples were compared with those in control samples to calculate the biases caused by interference. Given the particular mechanism of interference by hemolysis assumed in the case of insulin (i.e., degradation by IDE), the measurements were performed kinetically at different time intervals. The acceptance criterion for defining significant interference of hemolysis in insulin measurements was set at 10%. A point-to-point regression approach was used to estimate the degree of hemolysis, producing a significant interfering effect at each specific time point.

### 2.3. Evaluation of the Effect of 6bK on Insulin Levels of Serum Samples with and Without Hemolysis

A dose–response study was conducted to assess the effective dose of 6bK for inhibiting insulin degradation caused by hemolysis. Various concentrations of 6bK (up to 100 µM) were added to hemolyzed sera, and insulin was measured over a period of up to 7 days. Then, the protective effect of the selected dose of 6bK (10 µM) was evaluated in samples with mild, moderate, and severe hemolysis with serum insulin concentration ranging from 3 to 20 mUI/L. The 6bK was added to sera with induced hemolysis through hemolysate addition or to whole blood prior to hemolysis induction by vortexing. Insulin levels were also measured in parallel in the same hemolyzed samples without 6bK (vehicle, water) or bacitracin (700 µM), as well as in the same serum samples without induced hemolysis, with either vehicle or 6bK (10 µM).

### 2.4. Evaluation of the Effect of 6bK on C-Peptide, Proinsulin, and Standard Biochemical Parameters

The effect of 6bK on C-peptide, proinsulin, and standard biochemical parameters was assessed by comparing the concentration results in serum samples (n = 5) with added 6bK (10 µM) or vehicle. C-peptide and proinsulin measurements were performed on serum samples that were stored at −20 °C for 24 h and 10 days, respectively, after the addition of 6bK or vehicle. Standard biochemical parameters were measured on serum samples stored at room temperature for up to 24 h after the addition of 6bK or vehicle.

### 2.5. Statistical Analysis

The concentration results are expressed as mean ± SEM. Given the small sample size and the repeated-measures design, statistical comparisons were performed using the non-parametric Friedman test for repeated measures. Statistical analyses were performed using GraphPad Prism 8 (GraphPad Prism Software Inc., San Diego, CA, USA). Since only unidentified pools of residual serum or whole blood samples were used in this study, institutional review board approval was not required.

## 3. Results

### 3.1. Insulin Concentration Decreases over Time with Increasing Hemolysis

**Figure 1:**
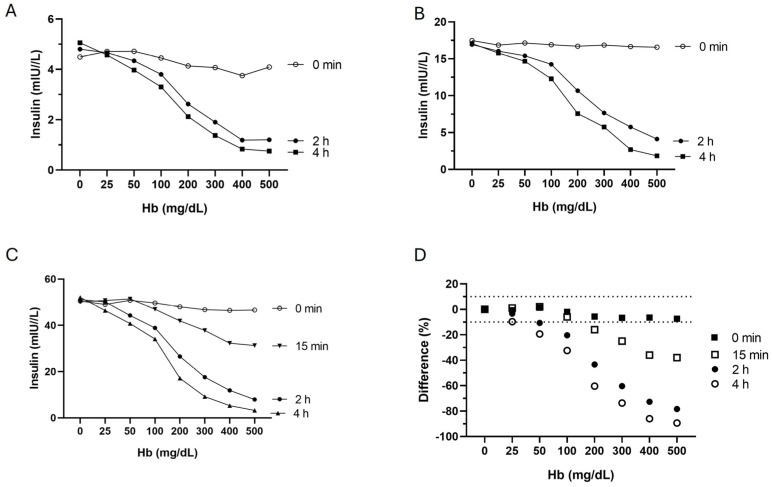
(**A**–**C**) Time-dependent effects of hemolysis on serum insulin levels at low, medium, and high baseline insulin concentrations. (**D**) Average insulin level changes (n = 3). Insulin concentrations remain unaffected by hemolysis at 0 min but significantly decrease after only 15 min in serum samples with ≥200 mg/dL of Hb.

As shown in Figure 1, the negative interference of hemolysis in insulin measurements at room temperature depended on both the duration and the level of hemolysate. While hemolysis at time 0 h (just after the hemolysate addition) did not significantly affect the concentration results of insulin, a significant decrease was observed after only 15 min in serum samples containing 200 mg/dL Hb. As calculated using point-to-point regression, insulin measurements were significantly interfered with by 140 (95% CI 132–148), 47 (95% CI 42–52), and 26 (95% CI 22–30) mg/dL Hb after 15 min, 2 h, and 4 h, respectively (Figure 1D).

### 3.2. 6bK Completely Reverses Hemolysis-Induced Interference in Insulin Measurements of Serum Samples

**Figure 2:**
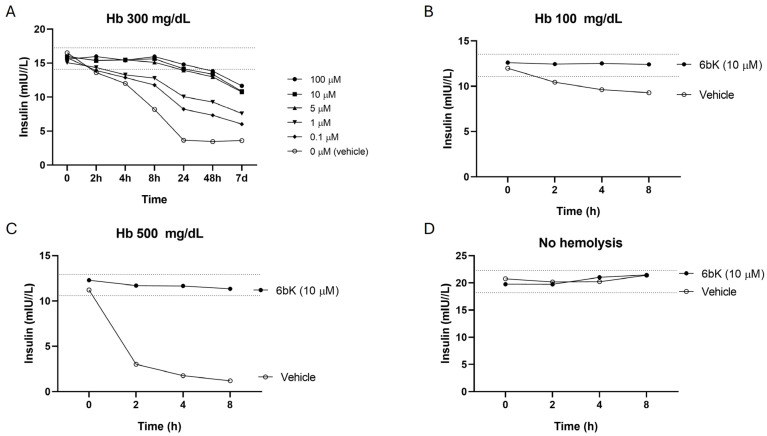
(**A**) Dose-dependent effect of 6bK on insulin measurements in hemolyzed serum samples with 300 mg/dL Hb (n = 3). (**B**,**C**). Effect of 6bK (10 µM) on serum samples with mild (Hb 100 mg/dL) and severe (Hb 500 mg/dL) hemolysis. (**D**) Effect of 6bK (10 µM) on non-hemolyzed serum samples.

**Figure 3:**
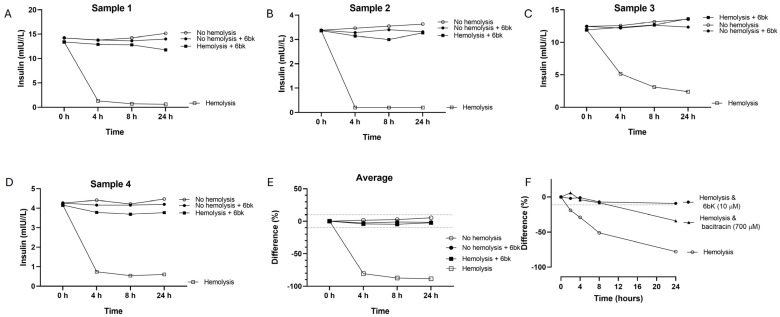
(**A**–**D**) Effect of 6bK (10 µM) on insulin measurements in four different whole blood severely hemolyzed (Hb > 500 mg/dL) and non-hemolyzed serum samples. (**E**) Average insulin level changes (n = 4). (**F**) Effect of 6bK (10 µM) and bacitracin (700 µM) on severely hemolyzed (Hb > 500 mg/dL) serum samples (n = 3).

The dose–response study showed that 10 µM of 6bK, added to a serum pool with moderate hemolysis addition (300 mg/dL Hb), effectively reversed the hemolysis-induced decline in insulin concentrations for up to 24 h (Figure 2A). The efficacy of 6bK was further confirmed in serum samples with mild (100 mg/dL Hb) and severe (500 mg/dL Hb) hemolysis (Figure 2B,C). Additionally, 6bK had no effect on the insulin concentration in non-hemolyzed samples (Figure 2D). Similarly, experiments using whole blood samples with severe hemolysis (>500 mg/dL) also showed that 6bK reversed hemolysis effects on serum insulin concentrations, with no impact in non-hemolyzed samples, for up to 24 h. No significant differences were observed in insulin concentrations between non-hemolyzed sera, non-hemolyzed sera with 6bK, and hemolyzed sera with 6bK, indicating a complete reversal of the interference (Figure 3E). As shown in Figure 3F, the effect of 6bK outperformed that of the non-specific IDE inhibitor bacitracin. Experiments performed with 6bK previously stored at room temperature for three weeks also showed a complete inhibition of hemolysis interference in insulin measurements (Figure S1).

The impact of hemolysis on insulin concentration was notably reduced at 4 °C. However, when hemolysate was added to the base serum and stored at 4 °C, a significant decrease in insulin concentrations was still observed (Figure S2A–C). Point-to-point regression analysis showed that insulin measurements were significantly interfered with by 185 mg/dL (95%CI 180–190) and 38 (95% CI 35–41) mg/dL Hb after 2 h and 4 h, respectively (Figure S2D). The addition of 6bK effectively reversed the effect of hemolysis at 500 mg/dL Hb (Figure S2F), with no significant effect on non-hemolyzed samples (Figure S2G).

### 3.3. 6bK Does Not Affect Standard Biochemical Parameters, C-Peptide, or Proinsulin Levels

**Figure 4:**
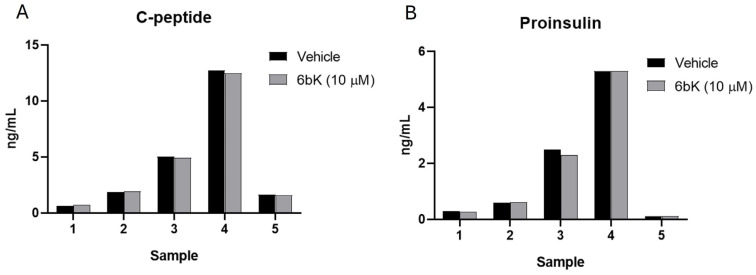
(**A**) C-peptide levels and (**B**) proinsulin levels in five non-hemolyzed serum samples treated with either vehicle (no 6bK) or 6bK (10 µM).

The presence of 6bK in serum samples did not affect the quantification of standard biochemical parameters typically included in biochemical assessments, such as ions, metabolites, proteins, and different enzymatic activities (Table 1). Additionally, no effects of 6bK were observed on the quantification of C-peptide or proinsulin (Figure 4).

## 4. Discussion

This study shows that the specific in vitro inhibition of IDE in serum and blood samples completely reverses the interference of hemolysis on insulin concentration, thereby determining the underlying mechanism of this interference. It also shows that using the selective IDE inhibitor 6bK is an effective strategy for accurately measuring insulin concentrations in both hemolyzed and non-hemolyzed clinical serum samples at standard laboratory conditions.

In agreement with previous studies [10,11], we observed that insulin concentration decreased in hemolyzed samples as a function of the amount of hemolysate and the duration of incubation. Importantly, this effect was not manifested at time 0 h, which is consistent with the kinetic degrading action of IDE and aligns with the previous observation that Hb alone does not cause interference [11]. However, we established that this effect occurs rapidly at mild–moderate hemolysis (after 15 min with hemolysates containing 200 mg/dL Hb or after 2 h with 50 mg/dL Hb). In routine clinical practice, these levels correspond to hemolysis typically classified as mild (approximately 20–100 mg/dL Hb) or moderate (100–300 mg/dL Hb), indicating that even samples generally considered acceptable for most clinical chemistry measurements (often up to ~300 mg/dL Hb) may already be affected. Similarly, Cook et al. [10] and Garinet et al. [11] observed a 10% decrease in insulin with hemolysates at only 100 mg/dL and 80 mg/dL Hb, as analyzed on the Roche Cobas 6000 and the Beckman Coulter Unicell DXI 800 (Brea, CA, USA), respectively. The similarity of our results, obtained using the IM1600 Atellica and the Roche Cobas insulin immunoassays, suggests that this interference is largely method-independent in current immunoassays that do not use insulin analogs and aligns with the enzymatic degradation of the endogenous insulin in vitro. However, as insulin immunoassays differ in antibody specificity and assay design, some variability across platforms in susceptibility to hemolysis-related interference, as well as in the effectiveness of 6bK, cannot be excluded, and further evaluation across additional analytical systems would be warranted.

Hemolysis is responsible for over 60% of blood sample rejections and is the most common preanalytical error in clinical laboratories [19]. This is particularly significant for insulin measurements, as neonatal samples and those from serial sampling studies, such as oral and intravenous glucose tolerance tests (OGTT and FSIVGTT), are highly prone to hemolysis [13,14]. For instance, hemolysis rates exceed 20% in samples drawn from peripheral intravenous catheters [20]. There are some proposed solutions to minimize the clinical impact of falsely low results of insulin measurements in hemolyzed samples. In the diagnostic evaluation of hypoglycemia, Cook et al. proposed that insulin results ≥ 3 mU/L may be indicative of inappropriate insulin secretion, regardless of the level of hemolysis [10]. Wu et al. proposed an equation for correcting insulin concentration results in hemolyzed samples that considers the time of hemolysis and Hb levels [21]. Although clinically useful under selected conditions, these approximations do not fully resolve the issue of interference, leaving significant uncertainty among clinicians and laboratory professionals on how to proceed with insulin measurements in hemolyzed samples. Thus, while the degree of hemolysis obtained by HIL measurements can provide useful information for managing results with other analytes [22], it has limited value for insulin due to the time and temperature dependency of the interfering effect. Despite this, many immunoassay manufacturers provide information about the effect of hemolysis on insulin determination based solely on the amount of Hb, which can easily be misinterpreted and lead to reporting insulin results that are highly interfered with. Of note, the vast majority of laboratories rely on information provided by manufacturers when establishing protocols for managing samples with hemolysis [23]. As demonstrated here, the time between extraction and analysis, as well as temperature, has a decisive impact on the interference. At room temperature, moderate hemolysis (~180–200 mg/dL Hb) induces significant interference within 15 min, while even mild hemolysis (~50 mg/dL Hb) becomes clinically relevant after 4 h. Although refrigeration at 4 °C delays this effect, it does not prevent it, with comparable interference observed after approximately 2 and 4 h, respectively. These findings underscore how rapidly interference can develop, even at low hemolysis levels, and indicate that prolonged storage should be avoided when insulin measurement is required, even under refrigerated conditions.

To prevent the effect of hemolysis on insulin degradation, we used 6bK, a specific IDE inhibitor with over 1000-fold selectivity for IDE over other metalloproteases and an IC50 of only 50 nM [16]. In a dose–response experiment, we identified 10 µM as the concentration that completely prevented the reduction of insulin levels in hemolyzed serum samples. This concentration aligns with the reported value that achieves a 100% IDE inhibition [16], suggesting that it can effectively inhibit IDE in clinical samples. This was further confirmed by adding 6bK (10 µM) to different serum and blood samples across a wide range of insulin concentrations and hemolysis levels. The results show that 6bK completely abolished insulin degradation under all the tested conditions. However, most experiments were performed using pooled serum or blood samples with artificially induced hemolysis. Although this approach allows for controlled evaluation of interference, further validation in naturally hemolyzed patient samples would strengthen the clinical applicability of these findings.

Previous studies employed non-specific IDE inhibitors to prevent the hemolysis effect, such as phydroxymercuribenzoate, p-chloromercuriphenylsulfonic acid, bacitracin, N-ethylmaleimide, 1,10-phenanthroline, EDTA, and diamide. These substances are limited by partial inhibitory effect, poor stability, or lack of IDE specificity. Among them, p-chloromercuriphenylsulfonic (0.4 mM) and diamide (5 mM) significantly reduced insulin degradation by IDE, although the insulin loss was not completely prevented [19]. More recently, bacitracin (700 µM) showed an attenuation of insulin degradation by hemolysis during insulin sensitivity testing [20]. In our experiments, bacitracin (700 µM) also attenuated the effect of insulin degradation in hemolyzed samples, although it did not achieve the complete inhibitory effect that was observed with 6bK at 70-fold less concentration (10 µM).

To further evaluate the clinical applicability of 6bK, experiments also involved adding it to multiple non-hemolyzed samples. In all cases, the insulin results were consistent with those from the same serum samples without 6bK. Similarly, the presence of 6bK did not impact the results of 18 commonly measured biochemical parameters in serum, including different enzymatic activities, nor did it alter the measurements of C-peptide or proinsulin, both structurally related to insulin and often measured together in clinical laboratories, suggesting no detectable off-target effects under the tested conditions. However, potential interference with other peptide hormones cannot be excluded, and additional studies would be required to fully assess long-term assay compatibility and possible matrix effects associated with 6bK use. Also, these findings were obtained in a limited number of serum samples and should be confirmed in a larger cohort, including samples from both healthy individuals and patients with different pathological conditions.

Despite the clear analytical advantages of 6bK for improving insulin quantification in hemolyzed samples, several translational aspects must be considered before routine clinical implementation. Integration into clinical workflows would require its incorporation into standardized blood collection tubes to ensure consistent preanalytical handling. However, given the added cost of 6bK, its widespread use may not be cost-effective in all scenarios, and its application may be better targeted to clinical settings with a high demand for reliable insulin measurements or an increased risk of hemolysis (e.g., neonatal samples or dynamic testing). Although 6bK demonstrated preserved activity after 21 days at room temperature under standard laboratory conditions, supporting its short-term practical applicability, further evaluation over longer storage periods will be required to confirm its stability for extended use in routine clinical settings. In addition, full analytical validation in accordance with in vitro diagnostic requirements will be necessary to demonstrate robustness, reproducibility, and compatibility with existing immunoassays. Finally, regulatory approval and successful implementation will likely depend on close collaboration with diagnostic manufacturers to enable integration into validated assay systems.

## 5. Conclusions

In conclusion, our study shows that specific inhibition of IDE by 6bK completely reverses hemolysis-induced insulin degradation in clinical samples, demonstrating the underlying mechanism of insulin measurement interference caused by hemolysis. The results also show that 6bK offers a novel solution for ensuring accurate insulin quantification in hemolyzed clinical samples under standard laboratory conditions.

## Figures and Tables

**Table 1:** Standard biochemical parameters measured in five different non-hemolyzed serum samples without 6bK (0 h) and after 2 h and 24 h following the addition of 6bK (10 µM).

Analyte	Range	Units	0 h	2 h	24 h	*p*
**Ions**						
Na	131–142	mEq/L	138 ± 2	137 ± 2	139 ± 2	0.14
K	3.9–4.5	mEq/L	4.1 ± 0.1	4.1 ± 0.1	4.2 ± 0.1	0.22
Cl	93–106	mEq/L	103 ± 3	101 ± 3	105 ± 2	0.11
Ca	8.4–9.2	mg/dL	8.8 ± 0.1	8.7 ± 0.2	9.0 ± 0.1	0.13
Mg	1.4–4.1	mg/dL	2.1 ± 0.5	2.1 ± 0.5	2.2 ± 0.5	0.30
**Metabolites**						
Glucose	68–195	mg/dL	112 ± 23	111 ± 23	114 ± 24	0.41
Creatinine	0.6–3.1	mg/dL	1.2 ± 0.5	1.2 ± 0.5	1.3 ± 0.5	0.39
Bilirubin	0.2–0.6	mg/dL	0.4 ± 0.1	0.4 ± 0.1	0.3 ± 0.1	0.27
**Proteins**						
Total proteins	54–76	g/L	67 ± 4	66 ± 4	67 ± 4	0.34
Albumin	31–46	g/L	39 ± 2	38 ± 3	39 ± 3	0.36
Reactive C-protein	<0.4–0.7	mg/dL	0.5 ± 0.1	0.5 ± 0.1	0.5 ± 0.1	0.38
**Enzyme activity**						
AST	19–121	U/L	64 ± 18	61 ± 20	64 ± 19	0.51
ALT	14–199	U/L	70 ± 34	69 ± 33	66 ± 31	0.45
GGT	14–311	U/L	126 ± 57	125 ± 57	128 ± 56	0.55
LDH	146–260	U/L	201 ± 22	200 ± 22	205 ± 23	0.37
Lipase	30–173	U/L	71 ± 26	70 ± 25	70 ± 25	0.61
ALP	40–258	U/L	127 ± 47	130 ± 47	134 ± 49	0.33
Amylase	61–122	U/L	79 ± 12	78 ± 12	81 ± 13	0.43

Data are shown as mean ± SEM. Statistical analysis was performed using the Friedman test for repeated measures.

## Data Availability

The dataset is available on request from the authors.

## Supplementary Materials

The following supporting information can be downloaded at: https://www.mdpi.com/article/10.3390/diagnostics16121927/s1, Figure S1: Effect of 6bK solution previously stored at room temperature for 21 days; Figure S2: Effect of hemolysis and 6bK treatment on insulin measurements at 4 °C.

## Abbreviations

The following abbreviations are used in this manuscript:

CLSI	Clinical & Laboratory Standards Institute
DSIVGTT	Frequently Sampled Intravenous Glucose Tolerance Test
HIL	Hemolysis, Icterus, Lipemia
HOMA-IR	Homeostasis Model Assessment of Insulin Resistance
IDE	Insulin-Degrading enzyme
OGTT	Oral Glucose Tolerance Test
QUICKI	Quantitative Insulin Sensitivity Check Index
