# Supplementary material for: Selective Inhibition of Insulin-Degrading Enzyme Eliminates Hemolysis Interference in Serum Insulin Measurements
Source: Diagnostics (Basel). 2026 Jun 22;16(12):1927. doi: 10.3390/diagnostics16121927 (PMC13297676; doi:10.3390/diagnostics16121927)
Supplement: Supplementary file 1 [file diagnostics-16-01927-s001.zip › diagnostics-4326230-supplementary.pdf]

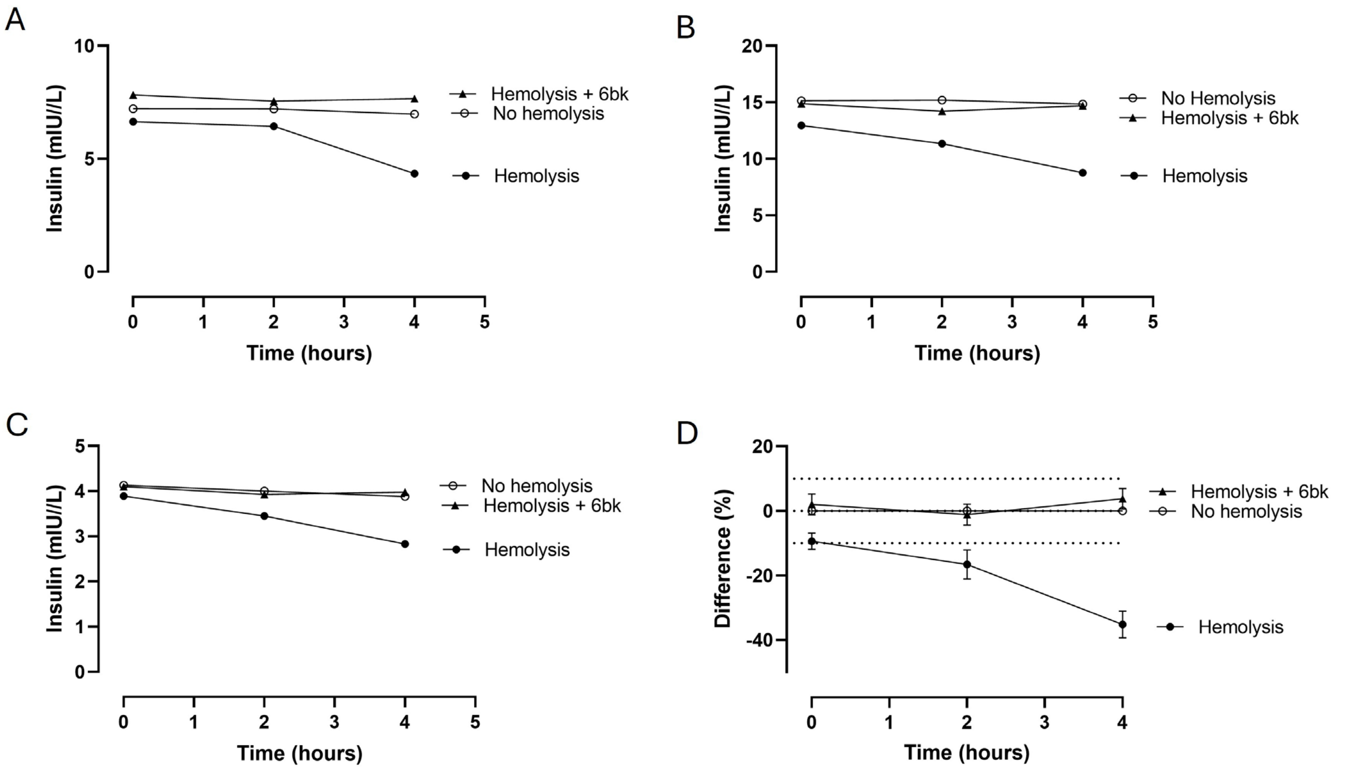

Supplementary Figure S1. Effect of 6bk solution previously stored at room temperature for 21 days. (A-C) Effect of 6bK (10 μM) on insulin measurements in three severely hemolyzed whole blood serum samples (Hb>500 mg/dL) at low, medium, and high baseline insulin concentrations (mean of two replicates). At 4 hours, hemolysis resulted in a negative bias of −35% (panel A), −39% (panel B), and −32% (panel C) compared to non-hemolyzed samples, whereas the addition of 6bk maintained values within +4%, −1%, and −2%, respectively. (D) Average insulin level changes (n=3). At 4 hours, hemolysis resulted in a negative bias of −35% compared to non-hemolyzed samples, whereas the addition of 6bk maintained values within ±5% of the control condition. The acceptance criterion for defining significant interference in insulin measurements was set at 10%. 6bK stock solutions (1 mM in water) were stored at room temperature in standard polypropylene tubes, protected from direct light exposure. No specific sterile conditions were applied beyond standard laboratory handling of analytical reagents.

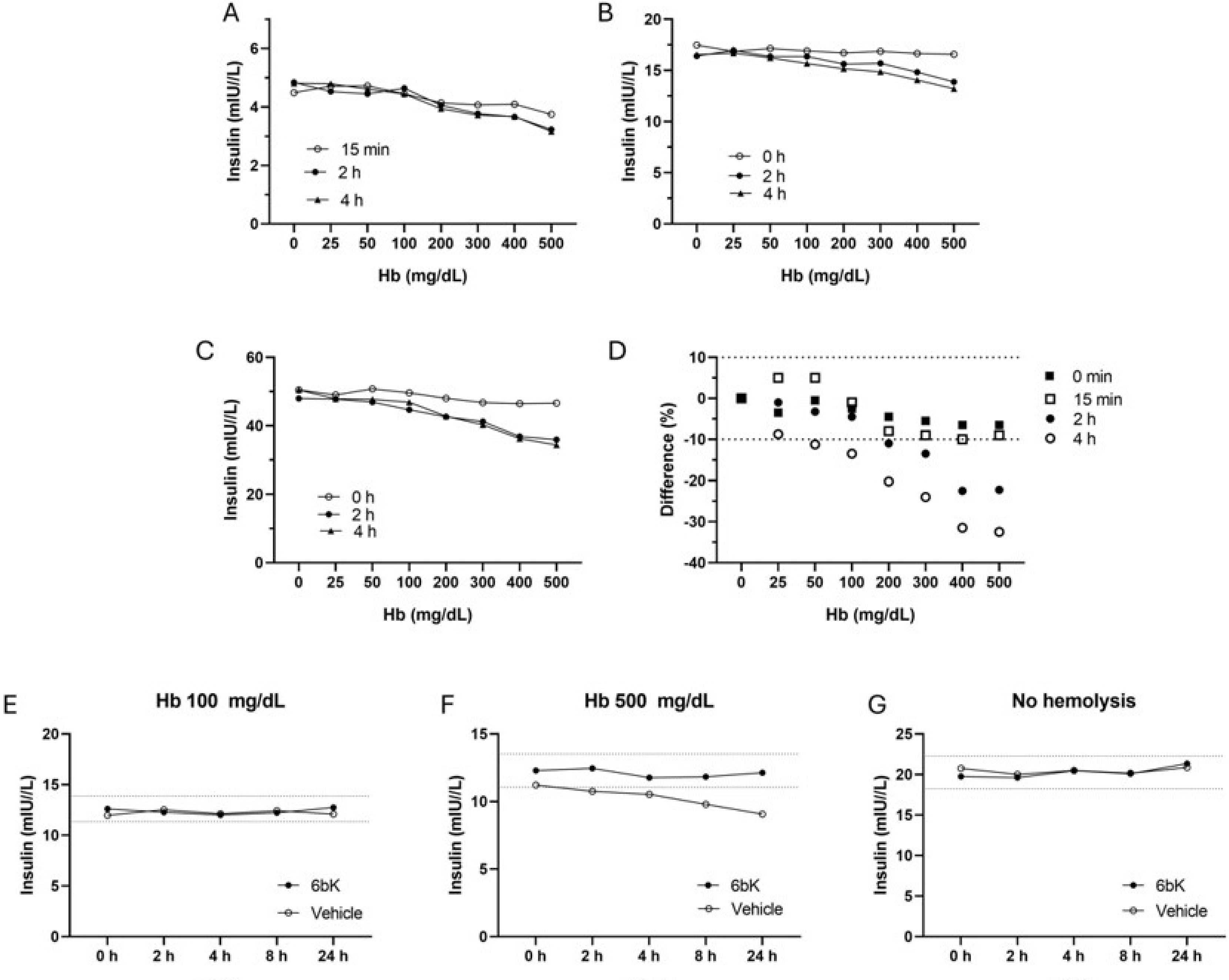

A
B
C
D
E
F
G
Insulin (mIU/L)
Difference (%)
Hb (mg/dL)
15 min
0 h
2 h
4 h
0 min
Hb 100 mg/dL
Hb 500 mg/dL
No hemolysis
6bK
Vehicle
0 h
2 h
4 h
8 h
24 h
0
25
50
100
200
300
400
500

Supplementary Figure S2. Effect of hemolysis and 6bk treatment on insulin measurements at 4ºC**.** (A-C) Time-dependent effects of hemolysis on serum insulin levels at low, medium, and high baseline concentrations, with samples stored at 4ºC (mean of two replicates). (D) Average insulin level changes (n=3). Insulin concentrations remain unaffected by hemolysis at 0 minutes but significantly decrease after 2 hours in serum samples with ≥50 mg/dL of Hb. . At 2 hours, hemolysis induced a negative bias of −5%, −12%, and −22% at Hb concentrations of 100, 300, and 500 mg/dL, respectively, while at 4 hours the corresponding biases were −13%, −24%, and −34%. (E-G) Insulin levels in serum samples with Hb 100 mg/dL, Hb 500 mg/dL, and in non-hemolyzed serum samples, treated with either vehicle (no 6bK) or 6bK (10 µM). All samples were stored at 4ºC, with total storage duration and time between hemolysis induction and insulin measurement of up to 4 h (Figures A–D) and up to 24 h (Figures E–G); no freeze–thaw cycles occurred. The acceptance criterion for defining significant interference in insulin measurements was set at 10%.
